# The Epithelial–Mesenchymal Transition at the Crossroads between Metabolism and Tumor Progression

**DOI:** 10.3390/ijms23020800

**Published:** 2022-01-12

**Authors:** Monica Fedele, Riccardo Sgarra, Sabrina Battista, Laura Cerchia, Guidalberto Manfioletti

**Affiliations:** 1 National Research Council (CNR), Institute of Experimental Endocrinology and Oncology “G. Salvatore” (IEOS), 80131 Naples, Italy; sabattis@unina.it (S.B.); cerchia@unina.it (L.C.); 2 Department of Life Sciences, University of Trieste, 34127 Trieste, Italy; rsgarra@units.it

**Keywords:** epithelial–mesenchymal transition (EMT), metabolism, cancer, tumor progression, Warburg effect, metabolic rewiring, breast cancer, lung cancer, thyroid cancer

## Abstract

The transition between epithelial and mesenchymal phenotype is emerging as a key determinant of tumor cell invasion and metastasis. It is a plastic process in which epithelial cells first acquire the ability to invade the extracellular matrix and migrate into the bloodstream via transdifferentiation into mesenchymal cells, a phenomenon known as epithelial–mesenchymal transition (EMT), and then reacquire the epithelial phenotype, the reverse process called mesenchymal–epithelial transition (MET), to colonize a new organ. During all metastatic stages, metabolic changes, which give cancer cells the ability to adapt to increased energy demand and to withstand a hostile new environment, are also important determinants of successful cancer progression. In this review, we describe the complex interaction between EMT and metabolism during tumor progression. First, we outline the main connections between the two processes, with particular emphasis on the role of cancer stem cells and LncRNAs. Then, we focus on some specific cancers, such as breast, lung, and thyroid cancer.

## 1. Introduction

The concept that epithelial cells can transform into mesenchyme has been known since the early 1980s [1]. Later, it was called epithelial–mesenchymal transition (EMT) to delineate its transient nature. In fact, the reverse process, called mesenchymal–epithelial transition (MET), is also possible, due to the high plasticity of the epithelial tissue that can transdifferentiate to a mesenchymal phenotype, partially or completely, and then return to the epithelial. During EMT, epithelial cells lose their junctions and baso-apical polarity, while they acquire a back-to-front polarity and the ability to migrate and invade surrounding tissues. Changes also occur in the cell shape due to cytoskeleton reorganization and new signaling programs with the acquisition of a spindle phenotype [2,3]. Different biological contexts contribute to the induction of such transformations. Depending on these, we can distinguish three types of EMT: type 1, which occurs during embryogenesis; type 2, which occurs during wound healing and fibrosis; type 3, which occurs in cancer and represents the first step toward its progression to the metastatic stage, due to the acquired ability to erode the extracellular matrix, migrate, and extravasate into the bloodstream [4]. An emerging concept is that a hybrid epithelial-mesenchymal state harbors a higher plasticity for metastasis [5,6].

EMT is associated with complex metabolic reprogramming, which supports the energy requirements of increased proliferation and/or motility, as well as the growth in a new hostile environment in the case of type 3 EMT. In fact, in all the phases of the metastatic process, the supply of nutrients can be limited, and cancer cells undergo various degrees of stress to which they adapt by modifying their metabolism, including the metabolism of glucose, lipids, amino acids (AA), and nucleotides. In fact, most cancer cells are more dependent on glycolysis than on mitochondrial oxidative phosphorylation (OXPHOS) for their energy production, even in the presence of oxygen, a phenomenon known as the Warburg effect [7]. This altered glucose metabolism, which enhances biosynthetic fluxes and antioxidant defense during rapid proliferation of cancer cells, is regulated by transcription factors (TFs) such as the hypoxia inducible factor 1 alpha (HIF-1α) that activates either glycolytic enzymes or glucose and lactate transporters while inhibiting OXPHOS [8,9]. Moreover, cancer cells enhance glutamine metabolism, pentose phosphate pathway, and the synthesis of fatty acids (FAs) and cholesterol. EMT-TFs can induce such metabolic changes. In addition, it has been shown that mutations in metabolic genes, can activate EMT, supporting the idea that EMT and metabolism are closely interrelated and that both are necessary for the complete progression of the tumor to the metastatic stage [10].

## 2. The Epithelial–Mesenchymal Transition in Cancer

### 2.1. Cellular and Molecular Changes

Epithelial cells are characterized by apical-basal polarity and the presence of tight junctions, adherent junctions, and desmosomes that allow the formation of layers that are positioned on the basement membrane through hemidesmosomes and constitute the epithelia, i.e., a permeable barrier that covers tissues and organs. Activation of EMT in cancer, as in other physio-pathological situations, entails loss of cell polarity, disruption of cell–cell junctions, and degradation of basement membrane resulting in cells that acquire mesenchymal characteristics with front–rear polarity, a reorganized cytoskeleton characterized by actin stress fibers, increased cell protrusions and motility, and, in addition, the ability to degrade extracellular matrix (ECM) enabling cells to invade [3,11]. These morphological alterations are the result of changes in gene expression. Typical changes that characterize the EMT and lead to the loss of the epithelial barrier function are the downregulation of E-cadherin together with other proteins that cause the destabilization and dissolution of the different type of epithelial junctions. These changes are accompanied by the expression of proteins promoting mesenchymal adhesion such as N-cadherin, vimentin, fibronectin, integrin α5β1, and proteases such as MMP2 and MMP9 that alter cell adhesion, cytoskeleton, cell polarity, and the ECM.

### 2.2. EMT Transcription Factors

Changes of gene expression occurring during EMT are orchestrated by master regulators, i.e., TFs that, in addition to regulate each other, coordinate a cascade of events leading to the repression of epithelial genes and the induction of mesenchymal genes [3]. These EMT-TFs include SNAIL (SNAIL1 and SNAIL2/SLUG), TWIST (TWIST1 and TWIST2), and zinc-finger E-box-binding (ZEB). SNAIL1 represses epithelial gene expression by binding to the E-box DNA sequences, cis-elements present in several promoter proximal regions of epithelial specific genes. For example, it binds to the E-box located in the regulatory regions of the E-cadherin gene and recruits the polycomb repressive complex 2 (PRC2) that operates post-translational modifications on histones resulting in repression of E-cadherin expression [12,13,14]. In addition, SNAIL1 activates the expression of genes that contribute to the mesenchymal phenotype [3]. Similar to SNAIL1, the basic helix-loop-helix (bHLH) transcription factor TWIST downregulates the expression of epithelial specific genes and activates the expression of mesenchymal genes; it represses E-cadherin and induces N-cadherin with mechanisms different from SNAIL1 through the recruitment of the SET8 methyltransferase [15]. ZEB1 and ZEB2 can also bind E-boxes repressing or activating transcription [3], but the co-factors recruited by ZEB are different from those used by SNAIL and TWIST. They include the CTBP repressor or the chromatin SWI/SNF remodeling complex member BRG1 [16], while transcriptional activation is often mediated by the co-activators p300/CBP-associated factors PCAF and p300 [17]. In conclusion, SNAIL, TWIST, and ZEB, by binding to the E-boxes present in several regulatory regions of epithelial and mesenchymal genes, can coordinately regulate the expression of genes that define the epithelial or mesenchymal phenotype.

Among the TFs involved in EMT, we should also mention the architectural TFs that belong to the high mobility group A (HMGA) family. In fact, increasing evidence, including some from our laboratories, have shown a pivotal role of HMGA in inducing stem-like state and metastatic progression through the activation of EMT-TFs and enhancement of the transcription of EMT-related genes [18,19,20,21,22,23,24]. In basal-like breast cancer (BLBC), for example, HMGA1 promotes migration and invasion in vitro as well as metastases formation in vivo, by regulating genes linked to the Wnt/β-catenin, Notch, and Pin1/mutant p53 signaling pathways [18]. Similarly, in colorectal cancer, HMGA1 promotes EMT and metastases by positively regulating Wnt/β-catenin signaling [24,25]. HMGA2, instead, is induced by the TGFβ signaling pathway during EMT, regulating the transcription of the EMT-TFs SNAIL, SLUG, and TWIST in the NMuMG cellular model [20].

### 2.3. Pathways Regulating EMT

How is the EMT process triggered? The growth of the primary tumor modifies the ECM and creates a tumor microenvironment (TME) in which there are stromal cells, such as cancer-associated fibroblasts (CAFs), and inflammatory cells (T-lymphocytes, macrophages, and myeloid-derived suppressor cells) that secrete a vast array of chemokines, cytokines, and growth factors that strongly influence cancer cells. Specific signals present in this milieu activate pathways that induce EMT in cancer cells by the activation of EMT-TFs. A central role is played by TGFβ, Wnt, Notch, and growth factor receptors. The TGFβ family (three TGFβ isoforms, two activins, and many bone morphogenetic proteins) has a prominent role in EMT; the binding to the TGFβ family receptors leads to receptor phosphorylation and activation of the SMAD complexes that translocate to the nucleus and bind TFs regulating the expression of a set of genes including those coding for EMT-TFs. The Wnt signaling pathway has a crucial role in the embryonic development of all animal species, in the regeneration of tissues in adult organisms, and in cancer [26]. The canonical Wnt pathway is activated upon binding of Wnt ligands to the Frizzled family of membrane receptors, leading to the release and stabilization of β-catenin from the GSK3β–AXIN–APC complex. β-catenin, then, moves to the nucleus and becomes part of a transcriptional complex embedding TCF (T cell factor) and LEF (lymphoid enhancer-binding factor) to promote a gene expression program, which includes the activation of EMT-TFs [27]. The Notch pathway is activated upon binding of the Delta-like or Jagged family of ligands to the four different isoforms of the Notch receptor (Notch1-4). This binding triggers a series of proteolytic cleavage events that culminate in the release of the active, intracellular fragment of the Notch receptor (Notch-ICD), that, with a direct route from the membrane to the nucleus, functions as a transcriptional co-activator in association with different binding partners and TFs [28]. Several growth factors, such as epidermal growth factor (EGF), fibroblast growth factor (FGF), insulin growth factor (IGF), hepatocyte growth factor (HGF), platelet-derived growth factor (PDGF), and vascular endothelial growth factor (VEGF) act through their cognate tyrosine receptor kinases. The binding triggers receptor dimerization followed by the stimulation of the kinase activity that phosphorylate the receptor and leads to the activation of the PI3K/AKT, ERK/MAPK, p38 MAPK, and JNK pathways, promoting cell growth and proliferation, as well as cell migration and motility via induction of EMT [11,29]. Inflammation and hypoxia conditions that are present in the TME can activate EMT as well. Several cytokines trigger the phosphorylation and activation of Janus kinases (JAKs) and signal transducer and activator of transcription proteins (STATs) that, following dimerization, foster the transcription of genes encoding EMT-TFs. Hypoxia can promote EMT through HIF1α, which activates the expression of the EMT-TFs TWIST and SNAIL1 [3,30,31]. Evidence coming from in vitro cell cultures and in vivo models suggest the presence of signaling cooperation and the convergence of these pathways on common targets during EMT. Functional crosstalk among the different pathways have been reported and include, for example, the cooperation between the TGFβ pathway with FGF-activated growth factor receptors [32], and the crosstalk of TGFβ with Wnt and Notch signaling achieved through SMAD complexes [3,11].

### 2.4. EMT, Chemoresistance, and Cancer Stem Cells

The EMT program is a highly dynamic process. Cancer cells that enter in the EMT process usually progress only partially toward the mesenchymal state. This means that cancer cells do not necessarily complete the process toward a fully mesenchymal state and that not all the cells within a tumor will progress to the same “partial” state, i.e., there are cancer cells within the same tumor that reside at the same time in a different state of this transition process, and this contributes to intra-tumor heterogeneity [33]. Epithelial cells that have acquired enough mesenchymal characteristics and initiate to disseminate will begin to produce factors, for example, TGFβ, creating an autocrine signaling loop that reinforces the mesenchymal state of the cells, even if, moving away from the primary tumor cells, they are no longer exposed to the EMT-inducing signals present in the TME [34]. During the metastatic process, cells that have acquired mesenchymal traits leave the primary tumor site, invade the bloodstream, and arrive at the metastatic site where they re-epithelize. Therefore, it appears that in this reverse process of MET their partial mesenchymal state endow them with a phenotypic plasticity necessary for successful metastasis formation [33,35]. It is noteworthy that the experimental activation of EMT confers many characteristics of cancer stem cells (CSCs) to carcinoma cells [36,37]. CSCs represent a small subgroup of cells in the tumor bulk with stem-like features, acknowledged to be responsible of tumor onset, maintenance, and relapse after therapy. Considering their ability to seed new tumors, it is possible that the EMT program activation is necessary not only for the physical dissemination of carcinoma cells to distant tissues, but also for entrance into the CSC state that enables the disseminated cells to serve as founders of metastatic colonies [36]. The identification of the EMT program as a source of cells with CSCs characteristics in combination with the notion that CSCs are more resistant to conventional chemotherapy and radiotherapy suggest the involvement of the EMT process in therapeutic resistance. Increasing evidence supports this association, including a strong correlation between an EMT-associated gene expression signature and treatment resistance [38,39,40]. Due to their key role, CSCs constitute an election target for innovative anticancer therapies. Studies on the connections between EMT, CSCs, and therapeutic resistance are needed to develop new strategies for cancer treatment. 

## 3. Interplay between EMT and Metabolism

### 3.1. From EMT to Metabolic Changes

Mesenchymal cells have different energy requirements than epithelial cells. Consequently, metabolic changes are associated with EMT. However, it is still unclear how EMT induces the metabolic reprogramming of cancer cells. In a bioinformatics analysis of public datasets, the increased expression of 44 metabolic genes was significantly associated with the expression of a mesenchymal phenotype in nearly 1000 cancer cell lines and they were upregulated upon EMT induction. Interestingly, some of the enzymes encoded by these genes, such as dihydropyrimidine dehydrogenase (DPYD), an enzyme involved in pyrimidine catabolism, were also necessary for EMT, suggesting that metabolic rewiring is required to complete the reprogramming orchestrated by EMT [10,41]. Other metabolic genes upregulated by EMT include those coding for glutaminase 1 (GLS1), enzymes of the glutathione metabolism, cytochrome P450, aldehyde dehydrogenase, and glucose transporter 3 (GLUT3) [10]. In colorectal cancer cells, caveolin 1 (CAV1) overexpression boosts aerobic glycolysis through HMGA1-induced GLUT3 transcription. Accordingly, CAV1 downregulation reduces glucose uptake and ATP yield while promoting AMPK/p53-mediated autophagy [42].

EMT can also downregulate the expression of some metabolic genes, which is the case of the following: (i) glycolytic enzyme fructose 1,6-biphosphatase (*FBP1*), whose loss due to repression mediated by SNAIL favors the uptake of glucose and the diversion of glycolytic carbons towards biosynthetic pathways in BLBC [43]; (ii) fatty acid synthase (*FASN*) and acetyl carboxylase (*ACC*), involved in lipogenesis [44]; (iii) nucleoside transporter, the loss of which reduces the absorption of the anticancer drug gemcitabine in the pancreatic cancer [45]; and (iv) pyruvate dehydrogenase kinase 4 (*PDK4*), whose loss have been shown to promote proliferation, tumorigenicity, motility, and invasion of hepatocellular carcinoma cells [46,47]. However, the effects of EMT on lipogenesis are controversial because there are other studies showing that in prostate cancer cells undergoing TNFα-induced EMT, FASN and lipogenesis are upregulated [48]. Similarly, in some cancers, such as breast cancer (BC) and non-small cell lung cancer (NSCLC), EMT has been shown to either activate glycolysis, by inhibiting phosphofructokinase [49] and cytochrome c oxidase [50], or induce a shift from glycolysis to OXPHOS, via downregulation of PDK4, leading to an overall increase in AA, especially glutamate [47]. Finally, in colon cancer cells, EMT induction by TGFβ1 causes the nuclear translocation of pyruvate kinase M2 (PKM2), which is pivotal in promoting EMT [51]. 

The SNAILl/phosphofructokinase platelet (PFKP) axis plays a critical role in the dynamic regulation of glucose flux between aerobic glycolysis and the pentose phosphate pathway (PPP) during EMT. Specifically, SNAIL1 regulates glycolytic activity via repression of PFKP, a major cancer isoform of an enzyme involved in the first rate-limiting step of glycolysis. This, in turn, switches the glucose flux towards PPP, potentiating cancer cell survival under metabolic stress and increasing the metastatic potential [49]. Moreover, in vitro studies have demonstrated that prostate cancer cells undergoing EMT were less addicted to glucose than epithelial-like cells but showed higher levels of aspartate and its downstream metabolites, which, in turn, could be contributors of EMT and tumor progression [52]. 

Finally, a link between EMT and metabolic changes during dormancy, where a metabolic rewiring could be based on the dynamic shift between EMT and MET, has also been suggested [10].

### 3.2. From Metabolic Changes to EMT

In contrast, as already mentioned, increasing evidence supports the role of metabolic reprogramming, including changes in glycolysis, glutaminolysis, mitochondrial, and lipid metabolism, in sustaining and completing EMT. 

Overexpression of phosphoglucose isomerase (PGI), a glycolytic enzyme secreted by cancer cells that converts glucose-6P to fructose-6P, stabilizes the EMT-TFs ZEB1 and ZEB2 via NF-κB activation and induces EMT [53,54], while suppression of its expression induces MET [55]. Other glycolytic enzymes involved in the induction of EMT are the aforementioned FBP1, and subsequent enzymes of the same pathway, i.e., aldolase A (ALDOA), which converts fructose 1,6 biphosphate into glyceraldehyde-3-phosphate and hydroxyacetone phosphate; glyceraldehyde-3P-dehydrogenase (GAPDH), which catalyzes the reaction from glyceraldehyde 3-phosphate to D-glycerate 1,3-bisphosphate; and the lactate dehydrogenase (LDHA/B), which converts pyruvate to lactate [10].

Regarding mitochondrial metabolism, recent work has highlighted its link with EMT as mutations in mitochondrial enzymes, such as some involved in the tricarboxylic acids (TCA) cycle, are common in aggressive cancers and are correlated with an EMT signature [56,57,58]. Among them are: (i) succinate dehydrogenase (SDH), which converts succinate to fumarate, (ii) fumarate hydratase (FH), which converts fumarate to malate, and (iii) isocitrate dehydrogenases (IDHs), involved in the oxidative decarboxylation of isocitrate to alpha-ketoglutarate. Loss-of-function mutations of the first two enzymes determine accumulation of the intermediate metabolites succinate and fumarate that can induce EMT via epigenetic regulation of miR-200 [59], while accumulation of a new metabolite, 2-hydroxyglutarate, is responsible for the induction of EMT following *IDH1* and *IDH2* heterozygous mutations, again affecting the activity of miR-200 [60]. More complex and still unclear is the role of the first enzyme of the TCA cycle, citrate synthase (CS), which seems to be endowed with a dual role in promoting or repressing EMT depending on the cellular context [61,62]. Similarly, the role of glutaminolysis on EMT is controversial: if on the one hand it induces EMT through the glutaminase GLS1 which activates the EMT-TF SNAIL1, on the other hand the expression of the mitochondrial glutaminase GLS2 has an opposite effect on the same SNAIL1 and inversely correlates with the prognosis in hepatocellular cancer [63,64]. Indeed, the levels of GLS2 are inversely associated with EMT even in BC cells where downregulation of GLS2 was correlated with reduced mitochondrial activity and glutamine independence even under low-glucose conditions, suggesting that cells induced to undergo EMT become independent from glutamine [65]. Accordingly, it has been recently shown that nutrient stress induced by glutamine deprivation leads to the induction of EMT in pancreatic ductal adenocarcinomas (PDAC), via upregulation of SLUG, and consequent PDAC progression [66].

Finally, regarding lipid metabolism, overexpression of acetyl-CoA synthases (ACSL1 and ACSL4) as well as stearoyl-CoA desaturase (SCD), induce EMT and correlate with poor prognosis in colorectal cancer [67]. Interestingly, the ACSL/SCD axis targeted by miR-19b-1 was later exploited by the same group in colorectal cancer as a promising therapeutic approach [68]. In general, increasing evidence indicates that high levels of FAs promote EMT [69,70,71]. Furthermore, accumulation of FAs, consequent to the upregulation of FASN-induced lipogenesis could serve for the generation of pro-tumorigenic signals that support EMT [72].

**Figure 1:**
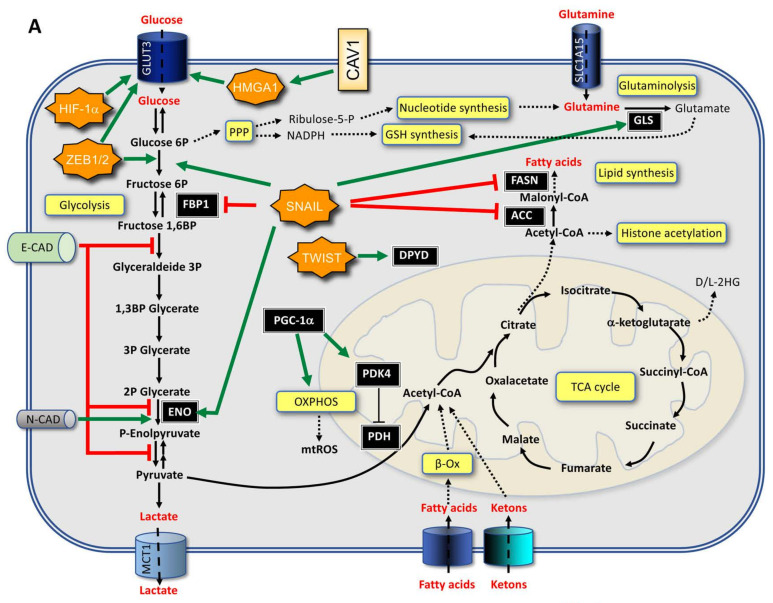

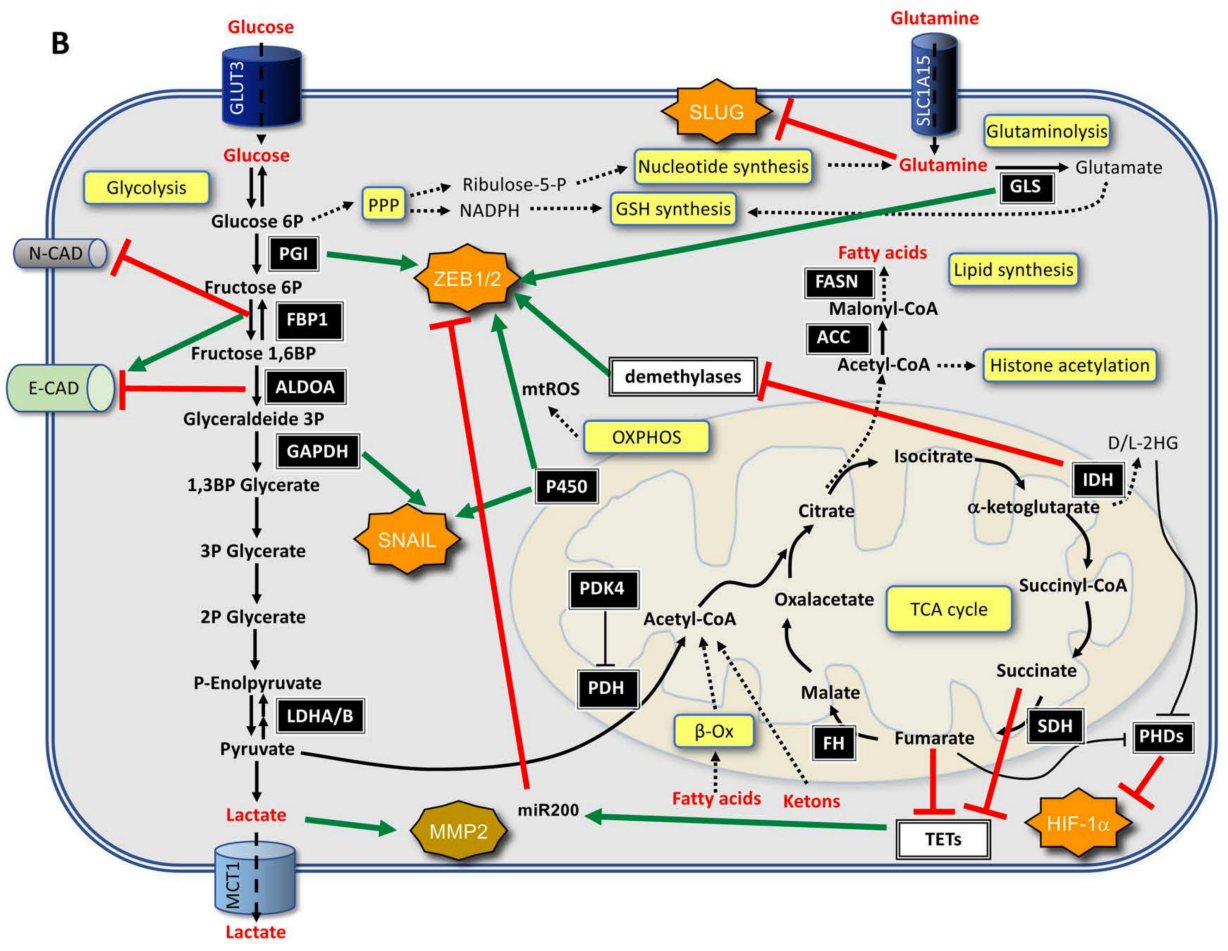
Schematic representation of the main mechanisms connecting metabolism and EMT in the cancer cell: (**A**) Effects of EMT markers on metabolic pathways; (**B**) action of metabolic mediators on EMT factors.

Overall, the link between EMT and metabolism is mutual and should be considered to be an interplay between the two processes that is functional to the progression and aggressiveness of the cancer (Figure 1). In particular, specific metabolic changes could lead to chromatin changes required for EMT-TF activity.

## 4. Role of EMT and Metabolism in CSCs

Unlike quickly proliferating tumor cells, CSCs are characterized by a certain quiescence, undifferentiated phenotype, ability to generate tumors in xenotransplantation, and evasion to standard chemotherapies. Whereas the old hierarchical theory claimed that CSCs are derived from life-long lasting tissue stem cells and, in turn, originate all the tumor cells of the tumor bulk, a new accredited theory holds that cancer cells (i.e., non-CSCs) can be converted in CSCs and vice versa, in an extremely plastic process [73], in which TME plays a central role, promoting de-differentiation and metabolic rewiring, with the ensuing gain of stemness features and EMT [74]. The TME space supporting the stem cell (the niche) provides a controlled environment for stem cell function, through the action of paracrine growth factors, cytokines, and niche cells, and regulates the self-renewal process by dictating the division mode (symmetric or asymmetric). In particular, the choice between symmetric and asymmetric division, which depends on mechanical signals transmitted by cell–cell and cell–ECM interactions to the stem cell polarity proteins [75], is extremely important in determining the stem cell fate [76]. Anchorage to the niche through cell–cell and cell–ECM interaction provides mechanical cues for the establishment of polarity signals necessary for asymmetric division, differentiation, and maintenance of tissue homeostasis; lack of these signals leads to undue symmetric division, increasing the pool of CSCs [77].

Stem cell and CSC EMT can be potently activated by mutations and inflammatory cytokines (TGFβ1, IL-6, TNFα, catecholamines, etc.) [74]. Even though CSC EMT has similarities with the one described for other non-stem cells, such as acquisition of migratory properties, and is mediated by the expression of the same EMT-TFs and architectural factors, such as SNAIL1, ZEB1, TWIST, and HMGA1, in CSCs, it implies some peculiar aspects, such as loss of contacts with the niche and promotion of tumor-initiating ability [73]. Studies from our laboratory have demonstrated that HMGA1 expression is enriched in the CD133+ subpopulation of colon CSCs [78] and affects Notch1 function by suppressing NUMB expression [79] and phosphorylation [80]. In BLBC cell lines, HMGA1 activates stemness and key migratory genes, related to the Wnt/β-catenin and Notch pathways [18]. However, even though a relationship exists between EMT and stemness, the two processes are not inextricably linked and the activation of TWIST1 in mammary cells can promote a long-lasting stemness, which is able to endure even after TWIST1 shut-down, concomitant to the appearance of an epithelial phenotype [81]. As highlighted in the previous sections, EMT is a reversible and transient process, so much so, that metastatic cells can display an epithelial phenotype; in addition, an effective and complete metastatic process foresees an EMT phase, allowing migration, and a reverse MET phase, where an epithelial phenotype is recovered to colonize the host tissue. This EMT-MET plasticity is part of a more extensive and complex CSC plasticity, through which CSCs and non-CSCs can be converted one in the other and vice versa [73], due to the action of key TFs; for example, a poised chromatin structure in the ZEB1 promoter allows non-CSCs to promptly adapt to changing microenvironmental cues, hence increasing their tumor-initiating ability [82]. Therefore, it looks like the capacity to transit back and forth between epithelial and mesenchymal phenotypes parallels the plastic nature of CSCs. 

Metabolic conversion represents a key step during the different phases of the metastatic process. Accordingly, inhibition of key glycolytic enzymes leads to the inhibition of CSC phenotypes, such as chemoresistance [83]. As highlighted in the previous sections, EMT and metabolism are functionally and bidirectionally interconnected, with this linkage playing a pivotal role in CSC physiology and tumorigenic potential [74]. Very often, EMT-TFs or co-regulators, also affect the expression of metabolic genes. This is the case of the chromatin architectural factors HMGA1 and HMGA2, which not only control the expression of key EMT players, such as SNAIL, E-CAD, and NUMB, but also regulate the metabolic phenotype through the expression of glycolytic mediators [84,85] or glucose transporters, such as GLUT3 [86], and take part into lipid metabolism through the activation of the c/EBPβ-PPARγ axis [87].

Alternatively, data from our laboratory have demonstrated that glucose reduction or caloric restriction-mimicking conditions shift brain tumor (glioblastoma) stem cells (BTSCs) towards an epithelial phenotype characterized by phospho-NUMB increased expression, supporting a bidirectional action between EMT and metabolism; this phenotype is reduced in HMGA1-high BTSCs [88], corroborating the role of HMGA1 as metabolic/EMT mediator.

Strikingly, metabolic rewiring also plays an active role in normal stem cells, as induced pluripotent stem cell (iPSC) reprogramming can be pursued by manipulating the metabolic pathways from OXPHOS to glycolysis [74,89], whereas embryonic stem cells and some adult stem cells activate epigenetic modifications, shifting from glycolysis to OXPHOS in order to differentiate [74,90]. However, different adult stem cells have specific metabolic preferences at the undifferentiated, differentiated, or proliferative state, without a fixed rule linking the stem cell state with a specific metabolic choice; rather, the type of metabolism is dependent on the type of stem cell. Similarly, in CSCs, metabolic reprogramming may affect both differentiation and self-renewal and, on the contrary, EMT can act as a master regulator of CSC metabolism. Proteins which are key player in EMT also directly affect the expression of metabolic genes. For example, ZEB1 regulates both EMT and metabolism, since its inhibition in pancreatic cancer cells impairs both EMT and the ability to undertake glycolysis [91]. SNAIL1 has been found to repress *FBP1* gene expression in BC cells, hence, promoting a pro-glycolytic metabolism [43]. 

Despite their large mitochondria endowment, CSCs embrace glycolysis as an elective source of energy and metabolites, yet do not disregard OXPHOS, glutamine, and FAs as alternative metabolic sources [74], being able to switch from one to another, depending on microenvironmental conditions, energy requirements [73], and the tissue of origin [92], by modifying the transcription of metabolic genes. Accordingly, very aggressive CSCs are able to metabolize FAs [92], necessary for cell membranes production, ROS inhibition, and drug resistance [74], and, appropriately, express the FA translocase CD36 for lipid uptake [93]. 

Targeting CSC metabolism holds therapeutic potentials; however, the heterogeneity and metabolic plasticity of the CSC population in the tumor bulk leaves unsolved the issue of the emergence of CSCs with different metabolic requirements, leading to relapse.

## 5. The Role of LncRNAs in EMT and Metabolism

The Human Genome Project revealed that the vast majority of our genome is transcribed and that non-coding RNAs (ncRNAs), i.e., RNA not translated into proteins, constitute a large portion of the transcriptome and are, however, functional in health and disease. NcRNAs are divided into two categories according to their size: (1) small ncRNA of less than 200 nucleotides, which include microRNAs (miRNAs), small nuclear RNA, small nucleolar RNA, tRNA derived small RNA, and Piwi-interacting RNA [94] and (2) long non-coding RNAs (lncRNAs) of over 200 nucleotides. The latter belong to a recently recognized class of RNAs involved in multiple physiological and pathological tumor activities [95]. Increasing evidence indicates that they play roles in both EMT and cancer metabolism, in particular glucose metabolism, suggesting they may be connectors of these processes. In some cases, such as for lncRNA MALAT1, H19, and ROR, a clear connection is in the upregulation of HIF-1α, which activates both TGFβ, involved in EMT induction, and PDK1, which inhibits the conversion of the pyruvate to acetyl-CoA, thus, inhibiting the TCA cycle. Mechanistically, lncRNAs often act as competing endogenous RNAs (ceRNAs) for miRNAs or through mediating epigenetic silencing by recruiting some components of the PRC2 [96,97]. As an example, lncRNA HULC (highly upregulated in liver cancer) functions as a ceRNA to mediate EMT by binding miR-200a-3p and, thus, upregulates ZEB1 [98]. Moreover, several lncRNAs interact with and participate in the function of PRC2, in particular by interacting with its associated subunit enhanced of zeste homolog 2 (EZH2), which plays a key role in EMT as it can silence E-cadherin promoter by H3K27 trimethylation [99]. In addition, lncRNAs can regulate glucose metabolism in tumor cells, favoring the aerobic glycolysis [100]. The list of lncRNAs involved in both EMT and Warburg effect include HOTAIR (HOX transcript antisense RNA); MALAT1 (metastasis-associated lung adenocarcinoma transcript 1, also called NEAT2); H19; UCA1 (urothelial cancer-associated 1); TUG1 (taurine-upregulated gene 1); PVT1 (plasmacytoma variant translocation 1); ANRIL (antisense non-coding RNA in the INK4 locus); CRNDE (colorectal neoplasia differentially expressed gene); ROR (regulator of reprogramming); CASC9 (cancer susceptibility 9); lincRNA-p21; Gas5 (growth arrest-specific transcript 5). Most of them promote both EMT and glycolysis, whereas some of them inhibit both processes (Table 1) [99].

## 6. EMT and Metabolism in Selected Cancers

### 6.1. Breast Cancer

#### 6.1.1. Glucose and OXPHOS Metabolism

Glucose concentration has a role in the regulation of different properties related to cellular aggressiveness; beside the increase in HK2 and pyruvate kinase, MCF-7 and MDA-MB-231 cells treated with high glucose concentrations display a higher migration speed and reduced F-actin production, a lower level of chromatin condensation, and are pushed towards an EMT process, as suggested by the rise of EMT markers such as vimentin [144]. The authors highlighted the fact that these in vitro experiments could mimic an in vivo situation related to high glucose plasma concentration, i.e., diabetes, which is a known risk factor for the onset and progression of cancer [144].

GLUT1 expression is downregulated by TGFβ1 stimulation in NMuMG, EpH4, MDA-MB-231, and MCF-7 cells, but this effect was related to the antiproliferative effect of TGFβ1 (72 h). Conversely, after a long time of exposure to TGFβ1, the expression of GLUT1 was partially restored. GLUT1 silencing partially impaired both cell proliferation and expression of the EMT marker vimentin in TGFβ1-stimulated NMuMG cells [145].

TGFβ1 ⇒ GLUT1 ↓ (72 h) GLUT1 ↑ (14 d)/GLUT1 (glucose uptake) ⇒ cell proliferation and EMT

BBT-474 and MCF-7 cells cultured for several weeks under 3D mammosphere forming conditions underwent a stable EMT. Compared to parental cells with an epithelial phenotype, mesenchymal-like BT-474 and MCF-7 displayed an enhanced aerobic glycolysis (higher glucose uptake, lactate production, glucose dependency for survival, and insensitivity to OXPHOS inhibition). Enhanced glycolytic flux was due to increased expression of glucose transporters (GLUT3 and GLUT1), lactate dehydrogenases (LDHA and LDHB), and monocarboxylate transporters for the lactate export (MCT2 and MCT4). MCT2 upregulation was due to STAT3 signaling [146].

A distinct glucometabolic phenotype is present in BC cell lines of different subtypes and different metastatic potentials. In particular, basal-like cell lines such as MDA-MB-231, have decreased mitochondrial respiration and increased lactate production as compared with luminal cell lines, such as MCF-7, indicating that OHPHOS deficiency and the switch to glycolysis are strictly correlated with the metastatic potential. Knockdown of specific EMT markers, such as E-cadherin and β-catenin, in MCF-7 cells causes a significant decrease in mitochondrial respiration and increased lactate production, implying that OXPHOS modifications are part of the metabolic reprogramming that occurs during EMT [147].

**Figure 2:**
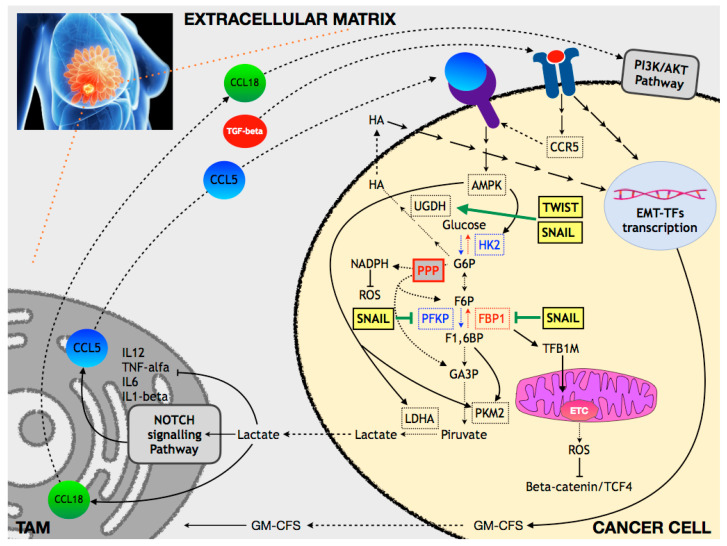
Overview of some of the main mechanisms connecting metabolism and EMT in BC.

Hyaluronic acid (HA), an ECM component, is a polysaccharide (up to 100,000 glycosyl groups) made by repeated glucuronic acid and acetylglucosamine disaccharides. The biosynthetic pathway starts from glucose-6P (G6P) and fructose-6P (F6P) that are two glycolytic metabolites. By crossing gene expression information with metabolomics data related to EMT, the group of Arun Sreekumar found that UDP-glucose dehydrogenase (*UGDH*) was among the upregulated genes together with hyaluronan synthase 2 (*HAS2*). UGDH has been found to have a prognostic value (relapse free survival) in ER-negative BC patients; interference with hyaluronic acid synthesis, both by siRNA-mediated silencing of UDGH expression or by the hyaluronic acid inhibitor 4-methylumbelliferone (4-MU), had a strong impact on the aggressive traits of MDA-MB-231 cells (invasion, sphere formation, and tumor formation in SCID mice). Interestingly, the authors demonstrated that, in TWIST-overexpressing HMLE cells (human mammary epithelial cells), there was an increase in the glucose turnover with a concomitant increased flux into UDP-glucuronic acid and UDP-N-acetylglucosamine, while the flux into citrate was unaffected, suggesting that for the execution of EMT there is an increase in the glycolytic flux that, however, is deviated towards lateral biosynthetic pathways (Figure 2) [148].

EMT ⇒ Glycolytic flux ↑, UGDH ↑, and HAS2 ↑ ⇒ HA production ⇒ ECM remodeling

Phosphoglucomutase 5 (PGM5) is one of the enzymes involved in the conversion of Glucose-1P (G1P) to G6P. PGM5 is downregulated in BC tissues as compared with adjacent normal non-cancerous tissues and suppresses proliferation and migration in different BC cellular models (MCF-7 and ZR75-1 cells). Importantly, PGM5 downregulation was responsible for an increase in the production of G6P, lactate, and ATP; thus, supporting the idea that the presence of PGM5 diverts glucose to glycogen formation [149].

PGM5 ↓ ⇒ Proliferation, motility, EMT ↑ and G6P, Lactate, ATP ↑

G1P ⇒ G6P ⇒ glycolysis

Overexpression of TWIST in the normal immortalized human mammary epithelial cell line MCF10A promotes migration and invasion along with increased glucose consumption and a higher rate of lactate production (glycolytic switch), as well as a decrease in mitochondria mass. Interestingly, interference with TWIST expression reverted this glycolytic metabolic switch. The metabolic alteration was indeed due to the alteration of the TWIST-dependent gene expression of several enzymes involved in glucose metabolism and, in particular, G6PD, PKM2, and LDHA. Dissection of the pathway involved in this metabolic rewiring (performed in MCF10A and BT549 cells) evidenced that, on the one hand, TWIST expression led to direct downregulation (at the promoter level) of p53 and this was related to the upregulation of G6PD that, in turn, fostered the activity of the PPP, providing NADPH for anabolism. On the other hand, upregulation of PKM2 and LDHA was due to β1-integrin/FAK-mediated activation of the PI3K/AKT/mTOR signaling pathway [150].

TWIST ⇒ p53 ↓ & β1 integrin ↑ // p53 ↓ ⇒ G6PD ↑/β1 integrin ↑ ⇒ PI3K/AKT/mTOR ⇒ PKM2 ↑ & LDHA ↑

As mentioned above, SNAIL is one of the main factors involved in EMT and it regulates the expression of key metabolic enzymes, such as PFK-1, FBP1, and ACC2. From the analysis of transcript abundance of *PFK-1*, *FBP1*, and *ACC2* within clinical samples collected by The Cancer Genome Atlas program, it emerged that the different intrinsic subtypes show different expression patterns related to these metabolic genes. In particular, it has been found that in luminal A and B subtypes, PFK-1 is downregulated, implying that the glycolytic flux is diverted towards PPP and that, due to the presence of FBP1, gluconeogenesis is active and DHAP and GA3P can be recycled to fuel the PPP. On the contrary, in the basal-like subtype, both FBP1 and ACC2 are downregulated, both glycolysis and PPP have the possibility to be fueled, but in this case, due to the suppression of ACC2, which catalyzes the formation of malonyl-CoA (a strong inhibitor of CPT1), there is a boost towards OXPHOS due to the availability of metabolites from both glycolysis and PPP (DHAP and GA3P) and FAs [151].

FBP1, the enzyme involved in the formation of F6P from fructose 1,6 biphosphate (F1,6BP) along the gluconeogenesis pathway, is repressed through DNA methylation by the binding of the SNAIL–G9a–Dnmt1 complex on its promoter in BLBC. It has been that FBP1 mRNA is expressed at a lower level in basal-like tumors than in other BC subtypes. Consistently, FBP1 protein is nearly undetectable in TNBC samples and BLBC cells as compared with luminal tumor samples and cell lines. Researchers have provided compelling data that the absence of FBP1 causes an increase in glucose uptake (via the MondoA/ChREBP/Mlx axis) and, in parallel, increased lactate generation and decreased oxygen consumption. The absence of FBP1 is responsible for an increase in F1,6BP, which is a modulator of PKM2 tetramerization, and hence, of its activation. In parallel, however, the expression of FBP1 is linked to that of TFB1M, a transcription factor involved in mitochondrial biogenesis and, therefore, in the expression of the components of the electron transport chain (ETC). As a consequence, FBP1 downregulation is associated with impaired mitochondrial activity (complex I activity), suppression of ROS production, and a related increase in β-catenin activity. In summary, BLBC cells enhance glycolytic flux and impair OXPHOS that is related to the SNAIL-mediated downregulation of FBP1. Importantly, FBP1 suppression is also linked to the ability of cancer cells to form tumorspheres and to the acquisition of CSC markers (Figure 2) [43].

SNAIL ⇒ FBP1 ↓ ⇒ F1,6BP ↑ ⇒ PKM2 ↑ (1) // FBP1 ↓ ⇒ OXPHOS ↓ (2) // (1) and (2) ⇒ lactate ↑ // (2) ⇒ ROS ↓ ⇒ β catenin activity ↑ ⇒ tumorigenicity (in vitro and in vivo) ↑

SNAIL is a key element during metabolic stress. Under glucose starvation, interference with SNAIL expression is responsible for an increase in cell death and reduced clonogenic and metastatic abilities. SNAIL suppresses aerobic glycolysis by directly repressing, at the promoter level, the expression of PFKP, with consequences on the production of a large number of molecules, including different AAs and lactate. The suppression of PFKP is responsible for the deviation of G6P into the PPP and, thus, for the production of NADPH, an antioxidant co-factor, the availability of which is linked to the survival of BC cells exposed to oxidative stress (Figure 2) [49].

SNAIL ⇒ PFKP ↓ ⇒ PPP ↑ ⇒ NADPH ↑ ⇒ survival to oxidative stress

Cancer cells produce pyruvate as a result of the aerobic glycolysis pathway and convert pyruvate into lactate, which is, then, exported to the ECM. Conditioned media from BC cells (MDA-MB-231) stimulates macrophages to assume a tumor-associated macrophage (TAM) phenotype and to foster the production of chemokines, in particular, CCL5. Lactate stimulates macrophages via the Notch signaling pathway. Lactate-activated macrophages secrete TGFβ1, which is responsible for the production of CCR5 (the CCL5 receptor) in cancer cells (MCF-7) and activation of the EMT program. In summary, cancer cells activate macrophages, which, in turn, activate the CCL5/CCR5 axis in cancer cells. MDA-MB-231 cells stimulated by lactate-activated macrophages have increased levels of glycolytic enzymes, in particular, HK2, PKM2, and LDHA. These changes are linked to the CCL5/CCR5 axis via the AMP-activated protein kinase (AMPK) signaling pathway. Interestingly, this circuit has been shown to be responsible for conferring cancer cells the ability to form metastasis when injected into nude mice. These data highlight a self-sustaining signaling loop between cancer cells and TAM that is centered on aerobic glycolysis and is involved in imparting aggressive properties to cancer cells (Figure 2) [152].

Cancer cells (aerobic glycolysis) ⇒ lactate ⇒ macrophages ⇒ TAMs (secreted CCL5 ↑ and TGFβ1 ↑ ⇒ Cancer cells (CCR5 ↑, EMT ↑, HK2 ↑, PKM2 ↑, LDHA ↑) ⇒ lactate (loop)

By investigating the relationship between mesenchymal-like cancer cells and macrophages, it has been demonstrated that cancer-derived lactate production was essential for cancer-cell mediated GM-CSF-dependent induction of the TAM phenotype that promotes tumor invasion and metastasis. This phenomenon is due both to the lactate-dependent abrogation of the secretion of proinflammatory cytokines (IL-12, TNFα, IL6, and IL1β) and to the induction of CCL18 secretion, which, in turn, stimulates cancer cell EMT. In light of the abovementioned data, this depicts a sort of self-sustaining loop, i.e., once the cells receive an EMT stimulus they are forced to a metabolism rewiring that sustains/contributes to the enhancement of the EMT itself (Figure 2) [153].

Mesenchymal-like cancer cells ⇒ GM-CSF ↑ ⇒ TAM ⇒ CCL18 ↑ ⇒ EMT in cancer cells

Mesenchymal-like cancer cells ⇒ Lactate ⇒ Proinflammatory cytokines↓

MCF-7 cells undergo EMT under a prolonged stimulation with TGFβ1 (0–9 days). TGFβ1 stimulation promotes an increase in ATP production that is due to an increased FAs oxidation, as revealed by the transcriptional upregulation of CPT1 and CD36, the downregulation of FASN, and an increase in mitochondrial ROS. These effects could be linked to the activation of the energy sensor AMPK [154].

TGFβ1 ⇒ EMT (FAs catabolism ↑, ATP ↑, and ROS ↑)

By using a PEGylated graphene oxide (PEG-GO) nanoparticle-based strategy to transport xanthohumol (PEG-GO@XN, xanthohumol (XN), an ETC complex I targeting mitocans) to the mitochondria, it has been demonstrated that impairment of OXPHOS and ATP production was linked to: (i) disruption of F-actin assembly, (ii) loss of cell migration and invasion abilities, and (iii) lower in vivo metastatic potential of BC cells (MDA-MB-231 and MDA-MB-436 cells). Simultaneously with this, the EMT process was compromised. Adequate functional OXPHOS was necessary for cancer cells to develop their aggressive potential [155].

ETC complex I targeting mitocans ⇒ OXPHOS and ATP production ↓ ⇒ EMT, migration, invasion, and metastatic abilities ↓

Microcalcification is one of the BC features exploited for cancer detection and has a prognostic value. BC cells (MDA-MB-468 and MCF-7) treated with an osteogenic cocktail: (i) form microcalcifications, (ii) display a higher metastatic potential (enhanced motility, migration, and invasion abilities) and (iii) undergo a gene expression rewiring towards the mesenchymal phenotype (EMT). Interestingly, within this context, OXPHOS metabolism is strongly enhanced. Indeed, the expression of PDHA (pyruvate dehydrogenase A) and all the five respiratory complexes (C-I to C-V) are upregulated upon osteogenic stimulation. A very interesting aspect is that the blockade of OXPHOS by rotenone does not affect the osteogenic differentiation but abrogates the EMT, implying that OXPHOS lies between osteogenic differentiation and EMT [156].

Osteogenic differentiation ⇒ Microcalcification ⇒ OXPHOS ↑ (PDHA ↑, C-I to C-V ↑) ⇒ EMT ↑

#### 6.1.2. Lipid Metabolism

PLD (phospholipase D) catalyzes the hydrolysis of phosphatidylcholine to generate phosphatidic acid, an important lipid secondary messenger. Interestingly, using cellular models of BC (MCF-7 and MDA-MB-231 cells), PLD expression has been shown to be under the control of a number of miRNAs (miRNA-203, -887, -3619, and -182), which, in turn, are regulated by E-cadherin (induction) and vimentin (suppression). In practice, the execution of the EMT program favors the activity of PLD that contributes to the migratory abilities of cancer cells [157].

The silencing of LIPH (lipase member H, lipase involved in the hydrolysis of triglycerides and phospholipids that generates FAs) has a strong impact on MDA-MB-231 cells. The loss of LIPH negatively affects the percentage of the CSC population, decreases the cellular aggressiveness (invasion and metastasis), and affects the oxygen consumption rate (OCR) [158].

LIPH ↓ ⇒ CSCs, EMT, invasion and metastasis ↓ & OCR ↓

SNAIL is responsible for the downregulation of ACC2 acting directly as a repressor at the promoter level. ACC2 is involved in the production of malonyl-CoA, a key metabolite for the fatty acid synthetic (FAS) pathway, but also a key inhibitor of CPT1 (carnitine-palmitoyl transferase 1), which is the rate-limiting enzyme for FA oxidation (FAO). In this way, SNAIL provides the cancer cells that live in a starved condition (due, for example, to a suboptimal blood circulation within the tumor mass) the possibility of obtaining ATP from FAO. Researchers have used different BC cell models (MCF-7 and MDA-MB-231 cell lines) to demonstrate that SNAIL expression is particularly relevant in glucose-starved conditions. As noted above, SNAIL also acts as a suppressor of PFK-1 [49], which is among the three rate-limiting enzymes of the glycolytic pathway (along with hexokinase and pyruvate kinase). By suppressing the expression of PFK-1, SNAIL diverts glucose into PPP, thus, providing NADPH, a key co-factor to fighting ROS.

It is noteworthy to mention that both the suppression of PFK-1 and ACC2 contribute to the availability of NADPH, since ACC2 is the first enzyme involved in FAS, which is a process that utilizes NADPH. However, it is not possible to forget that SNAIL inhibits the expression of FBP1, thus, impairing gluconeogenesis and facilitating the glycolytic pathway [43]. It has also been evidenced that the suppression of FBP1, in conjunction with FPK-1 can prevent the recycling of PPP products and foster their channeling towards the lower glycolytic pathway, thus, providing an energetic advantage in a glucose-starved condition. However, some discordances are evident. For example, the fact that when SNAIL-mediated suppression of FBP-1 was described, it was also associated with a suppression of ETC [43], which is in contrast with the evidence provided by the Kim and Yook groups [49,159] but this could be due to the different availabilities of glucose [159].

SNAIL (glucose starvation) ⇒ ACC2 ↓ ⇒ FAS ↓ (NADPH not consumed), FAO ↑

KDM5B is a lysine demethylase that is overexpressed in a variety of tumors and interference with its expression or with its activity with a specific inhibitor are linked to the downregulation of several EMT markers in BC cells (MCF-7 and MDA-MB-231). KDM5B is directly involved in the expression of the two enzymes of the FA biosynthetic pathway, FASN and ACLY. FA synthesis is related to the requirements of actively proliferating cells, both from an energetic and biosynthetic point of view [160].

SLC27A4 (solute carrier family 27, A4 member) is an FA transporter that is also characterized by acyl-CoA synthetase activity. Silencing of SLC27A4 expression in BC cells (Hs578T and MDA-MB-231) has an effect on FA uptake, as well as a modest effect on cell growth and cell cycle distribution, but a more prominent effect towards cell migration and invasion (impairment) and on the expression of factors involved in EMT (in particular as regards MDA-MB-231 cells: a decrease in vimentin, SLUG, N-cadherin, and α-SMA; however, an increase in E-cadherin). These data suggest that the disturbance of FA metabolism causes an alteration of the mesenchymal phenotype of BC cells [161].

SLC27A4 ↓ ⇒ motility ↓, invasion ↓, mesenchymal factors ↓ (vimentin, slug, N-cadherin, α-SMA)

In a mammosphere-based EMT model (MCF-7 adherent cells versus MCF-7 cultured in mammosphere forming conditions) the induction of the EMT program has been associated with relevant changes in cell lipidome. In particular, MUFA (monounsaturated FAs) were enriched and PUFA (polyunsaturated FAs, such as DHA) were strongly decreased in sphere cells, i.e., in those cells that underwent EMT. A parallel transcriptomic analysis showed that SCD (stearoyl-CoA desaturase), an enzyme involved in the formation of oleic acid, was upregulated, while several enzymes involved in PUFA formation were downregulated, among which included ELOVL2 (elongation of very-long-chain-fatty acids-like 2). The silencing of ELOVL2 in BC cells (MCF-7) fosters the acquisition of a higher motility and colony formation ability. Interestingly, silencing of ELOVL2 causes an upregulation of SREBP1 and SREBP2 (sterol regulatory element-binding 1/2), which is a key factor involved in regulating lipid biogenesis pathways [162].

EMT ⇒ SCD ↑ and ELOVL2 ↓ ⇒ MUFA ↑ and PUFA ↓ ⇒ DHA ↓ ⇒ SREBP1/2 ↑ ⇒ lipid biogenesis ↑

CAFs and cancer cells have a mutual influence. CAFs, by means of different secreted factors (HGF, TGFβ, and bFGF) can promote the acquisition of mesenchymal markers and enhanced membrane plasticity, thus, contributing to EMT in cancer cells. Interestingly, cancer cell migration has been linked to the activity of SCD1, since oleic acid is a MUFA that contributes to the plasticity of cell membrane [163,164]. Indeed, both the silencing and the pharmacological inhibition of SCD1 are responsible for a strong reduction in cell migration abilities and the supplementation with oleic acid, in both cases, can rescue the migratory impairment.

CAF ⇒ secreted HGF, TGFβ, and bFGF ⇒ BC cells: SCD1 ↑ ⇒ Oleic acid ↑ ⇒ migration ↑ (contribution to EMT)

Comparing MCF-7 with MDA-MB-231 cells as a model for EMT, it has been noted that MDA-MB-231 cells expressed higher level of key glycolytic factors (i.e., GLUT1, LDHA/B, and HK2) and that were free of key enzymes involved into FA biosynthesis. Indeed, MDA-MB-231 cells are strongly dependent on lipid availability due to their impairment of lipogenic enzymes (i.e., FASN, citrate carrier (CIC), ACLY, ACACA, and acetyl-CoA carboxylase A/B (ACACA/B)). The inability of MDA-MB-231 cells to synthesize FAs was compensated by an increase in the expression of factors involved in FA management, such as fatty acid-binding protein 5 (FABP5), CAV1, and perilipin-3 (PLIN3). These peculiar metabolic features of MDA-MB-231 cells make them strongly dependent on the availability of exogenous lipid supply. Moreover, MDA-MB-231 cells have a higher beta-oxidation rate with respect to MCF-7 cells, which highlights the importance of this energetic pathway for these cells [165]. 

The mevalonate pathway is central for the biosynthesis of cholesterol and coenzyme Q, for protein prenylation, and for the synthesis of dolichol, which is involved in the docking of oligosaccharides on the inner part of the endoplasmic reticulum (ER) for protein N-glycosylation. Therefore, it is evident that this pathway has a profound impact on cell energetics, signaling, and protein post-translational modifications. Statins, inhibitors of the mevalonate pathway, decrease the risk of BC recurrence after surgical removal of the tumor. It has been noted that BC epithelial cells (MCF10A) induced to undergo EMT by overexpression of SNAIL became sensitive to fluvastatin and that the sensitization was due to the interference of fluvastatin with dolichol synthesis. Researchers have demonstrated that EMT was associated with the upregulation of several N-glycans structures and that fluvastatin was responsible for their downregulation. In an in vivo mouse model, the researchers have shown that after primary tumor implantation, growth, and surgical resection, the treatment of mice with fluvastatin was able to strongly reduce metastasis formation and to prolong overall survival, highlighting the role of protein N-glycosylation in cancer metastasis in the context of cells that underwent EMT [166].

SNAIL ⇒ EMT ⇒ dolichol ↑ ⇒ N-glycans structures ↑

Mevalonate ⇒ olichol synthesis ↓ ⇒ N-glycans structures ↓

#### 6.1.3. Amino Acid Metabolism

Glutamine (Gln) is a key AA because of its contribution to different metabolic processes, such as nitrogen supply, a source of carbon atoms, nucleotide biosynthesis, and participation in the redox balance. Interestingly, Gln is also important for maintaining cellular stemness features in BC cells (MCF-7 and MDA-MB-231 cell lines) and its deprivation pushes them towards an epithelial phenotype, as assessed by changes in the expression levels of several mesenchymal and epithelial markers (E-cadherin, N-cadherin, and claudin-1) and their intracellular distribution [167]. 

BC cells are addicted to cystine to counteract the induction of canonical necrotic death. Several basal BC cell lines (i.e., MDA-MB-231, MDA-MB-157, BT-20, and others) undergo necrosis upon cystine deprivation, which is promptly rescued either by administration of a ROS scavenger (Necrox-5) or by inhibiting members of the canonical necroptotic pathway, i.e., RIPK1 (receptor interacting kinase 1) and MLKL (mixed-lineage kinase domain-like). Regarding the involvement of EMT in this phenomenon, the expression of EMT inducers (SNAIL1 and TWIST) in the cystine-independent luminal T47D BC cell line induces EMT and makes these cells sensitive to cystine deprivation. Basal BC with a mesenchymal phenotype has an activated MEKK4/p38/Noxa pathway that is responsible for mitochondria dysfunction and, consequently, for the generation of ROS. Cystine is required for the ROS detoxification process, since it is a key component of glutathione (GSH), one of the main endogenous scavengers of ROS [168].

#### 6.1.4. ROS Metabolism

GPX8 (glutathione peroxidase 8) is a metabolic factor essential for EMT. GPX8 is a member of the glutathione peroxidase family (GPX1-8) that does not use GSH but ER- resident reduced proteins (-SH groups) for the elimination of H_2_O_2_ produced by ERO1α oxidoreductase during disulfide bond formation onto PDI (protein disulphide isomerase, a relevant factor for protein folding, i.e., for disulphide bond formation) in the ER. GPX8 is overexpressed in basal B BC cell lines and its expression is strongly increased during TWIST-mediated induction of EMT in human mammary epithelial cells; interference with GPX8 expression in MDA-MB-231 cells causes MET, as evidenced by the acquisition of an epithelial morphology and by the loss of mesenchymal markers, migration capacity, and cancer stemness. GPX8 is essential for the IL6/JAK/STAT3 signaling pathway that has a relevant role in EMT. In particular, GPX8 is essential for the production of a functional form of the soluble IL6 receptor (sIL6R), which is responsible for the phosphorylation (i.e., activation) of STAT3 [169].

GPX8 ⇒ protein folding ↓ (disulphide bonds formation) ⇒ sIL6R (functional) ⇒ IL6/JAK/STAT3 pathway activation ⇒ EMT

### 6.2. Lung Cancer

#### 6.2.1. Glucose and OXPHOS Metabolism

**Figure 3:**
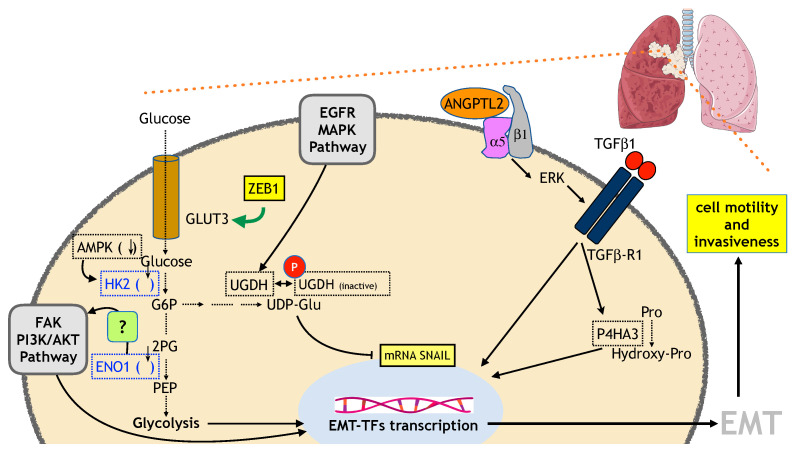
Overview of some of the main mechanisms connecting metabolism and EMT in lung cancer.

AMPK is an energy sensor kinase significantly involved in metabolism regulation. Interestingly, low AMPK expression levels correlate with tumor grade, TNM stage, and lymph node metastasis. Low AMPK levels in human bronchial epithelial HBE cells are responsible for an increase in glycolytic flux (due to the induction of HK2 expression which, in turn, promotes EMT (Figure 3) [170].

AMPK ↓ ⇒ HK2 ↑ ⇒ Glycolysis ↑ ⇒ EMT

GLUT3 (SLC2A3), a glucose transporter mainly expressed in neurons is expressed in NSCLC cells and, in particular, in those with mesenchymal characteristic (SW1573, H23, H450, A549, and Calu-6). GLUT3 expression is associated with the EMT induction by TGFβ stimulation in epithelial-like H2122 and H727 NSCLC cells and is under the transcription control of EMT-TFs, in particular ZEB1. GLUT3 expression is responsible for an increase in glucose uptake (Figure 3) [171].

ZEB1 ⇒ GLUT3 ↑ ⇒ Glucose uptake ↑

Angiopoietin-like protein 2 (ANGPTL2) is a secreted factor that can have both autocrine and paracrine effects and it is involved in cell motility and invasiveness. In different lung cancer (LC) cellular models (NCI-H460, NCI-H460-LNM35, NCI-H1975, and HCC15), ANGPTL2 is responsible for an increase in GLUT3 expression (and an increase in glycolytic flux) via an integrin α5β1/ERK-dependent pathway impinging on TGFβ and its downstream factor ZEB1, concurrently with the execution of the EMT (Figure 3) [172].

ANGPTL2 → integrin α5β1→ERK ⇒ TGFβ ↑ ⇒ ZEB1 ↑ ⇒ (GLUT3 ↑ & EMT)

In a very intriguing study, it was demonstrated that the mRNA stability of SNAIL1 was dependent upon the concentration of UDP-glucose (UDP-Glu) (high UDP-Glu ⇒ lower SNAIL1 mRNA stability), which, in turn, is under the control of UDP-glucose dehydrogenase (UGDH) (i.e., conversion of UDP-Glu into UDP-glucuronic acid). Indeed, the binding of HuR, a mRNA stabilizing protein, to SNAIL1 mRNA is negatively regulated by UDP-Glu, whose concentration is under the control of UGDH. Interestingly, UGDH is phosphorylated in an EGFR/MAPK-dependent way at tyrosine 473 [pUGDH(Y473)] and this drives its association with HuR. A correlation exists between levels of pUGDH(Y473) and patient survival/metastatic recurrence. Patients with lower survival and higher metastatic recurrence are characterized by a higher level of pUGDH(Y473) that implies a lower level of UDP-Glu, higher stability of SNAIL1 mRNA, and a boost towards EMT (Figure 3) [173].

EGFR/MAPK → UGDH ⇒ UDP-Glu ↓ ⇒ HuR/SNAIL1 mRNA ↑

UDP-N-acetylglucosamine (UDP-GlcNAc) is the substrate for O-linked N-acetylglucosamine transferase (OGT) that catalyzes the O-GlcNAcylation of proteins at serine/threonine residues. Silencing of OGT has an impact on several EMT markers [174]; in particular, it impairs N-cadherin, vimentin, and fibronectin expression upon TNF/TGFβ stimulation. The pathway for the synthesis of UDP-GlcNAc is involved in EMT in LC cells. Indeed, several enzymes involved in UDP-GlcNAc production have been found to be upregulated in TNF- and TGFβ-stimulated A549 spheroids; glutamine-fructose-6-phosphate transaminase 2 (GFPT2) was identified as the one with the higher fold change. *GFPT2* is under the direct regulation of NF-κB (positive) and sirtuin 6 (negative) and the interference with its expression impairs migration of mesenchymal cells [174].

TNF/TGFβ → NF-κB ⇒ GFPT2 ↑ ⇒ cell migration and invasion

Enolase (ENO1) overexpression is responsible for an increase in the expression level of SNAIL, N-cadherin, and vimentin (VIM); for the downregulation of E-cadherin (CDH1); and for an increase in LDHA and lactate production. In addition, these modulations are due to alterations in the FAK–PI3K–AKT axis (phosphorylation status/activation) and the functional outcome is a modulation of cell migration and invasion (cellular models, NSCLC cell lines A549 and SPCA-1) (Figure 3) [175].

ENO1↑ ⇒ FAK-PI3K-AKT ↑ ⇒ SNAIL, CDH2, VIM ↑ & CDH1 ↓

In LC cellular models forced to undergo EMT by prolonged TGFβ exposure, cells become resistant to targeted therapies and display a shift from glycolysis to OXPHOS. The mechanism underlying this phenomenon is the downregulation of PDK4, a kinase involved in the deactivation of pyruvate dehydrogenase (PDH). Alteration of PDK4 expression (either by overexpression or silencing) has a direct influence on the EMT process [47].

TGFβ ⇒ PDK4 ↓ ⇒ PDH ↑ ⇒ TCA ↑

In LC A549 cells, TGFβ regulates genes involved in mitochondrial function and biogenesis through the suppression of PGC-1α (peroxisome-proliferator receptor (PPAR) co-activator). Genes downstream of PGC-1α and involved in mitochondrial metabolism (*NRF2*, *PPAR-γ,* and *ERR-α*) are downregulated as well as their target genes (i.e., *SOD2*, *CytoC*, and *MCAD*), which play a direct role in ROS detoxification and mitochondrial metabolism [176].

TGFβ ⇒ PGC-1α ↓ ⇒ NRF2, PPAR-γ, ERR-α ↓ ⇒ SOD2, CytoC, MCAD ↓

Fyn-related kinase (FRK) is a member of the Src kinase family that is involved in cancer. FRK protein expression level is a prognostic factor in LC patients. FRK knockout in LC H1299 cells has a strong negative impact on glucose metabolism and indeed the Warburg effect is suppressed (inhibition of glucose uptake and acetyl-CoA and lactate production) along with general energy metabolism [177].

#### 6.2.2. Amino Acid Metabolism

TGFβ stimulated A549 cells display an increase in aspartic acid, glutamic acid and lysine levels, and a reduction in alanine, asparagine, citrulline, glutamine, glycine, histidine, hydroxyproline, isoleucine, leucine, phenylalanine, proline, threonine, and tyrosine. This effect is also reciprocal, since the depletion of most of these AAs in cell culture induces an EMT-like phenomenon. From the integration of metabolomic and genomic data, four genes involved in AA metabolism have been found to be upregulated in TGFβ-stimulated cells, i.e., arginase 2, glutaminase, alanyl aminopeptidase, and prolyl 4-hydroxylase subunit α 3 (P4HA3). P4HA3 has been found to be essential for TGFβ-induced AA changes and for the TGFβ-induced EMT process. According to the association between EMT and cellular aggressiveness, P4HA3 expression level has been associated with a poor prognosis and has been shown to play a relevant role in cell migration and metastatic abilities of cancer cells both in vitro and in vivo.

P4HA3 catalyzes the conversion of proline to hydroxyproline, thereby, reducing the proline level (Figure 3). Researchers have also highlighted a link between the urea cycle and the EMT process, hypothesizing that a decrease in the AA levels could be due to a metabolic reprogramming of the urea cycle [178].

TGFβ ⇒ P4HA3 ↓ ⇒ Proline ↓

TGFβ ⇒ AA ↓ (Urea Cycle ↑)

In A549 LC cells, SNAIL is responsible for the induction of EMT and concomitant metabolic reprogramming. Nucleotide biosynthetic intermediates are reduced, TCA intermediates remain unchanged, while those of the glycolytic pathway are downregulated. 3PG is the first glycolytic metabolite whose level is not affected by SNAIL. It is interesting that 3PG is the starting point of the serine and glycine biosynthetic pathway, and that glycine is a key molecule involved in folate production. Glycine decarboxylase (GLDC) is downregulated by SNAIL, which strongly affects the utilization of the glycolytic metabolites to generate folate via the serine/glycine metabolic shunt to sustain the anabolic processes involved in cell proliferation, in accordance with the shift “proliferation to migration” during EMT [179].

SNAIL ⇒ GLDC ↓ ⇒ Folates↓ (proliferation ↓) ⇒ glycolysis ↑ (migration ↑)

SLC38A3 is a transmembrane AA transporter (mainly glutamine, histidine, alanine, and asparagine) that mediates the Na^+^/AA export/H^+^ import. SLC38A3 is overexpressed in NSCLC as compared with normal tissues and is even more highly expressed in metastatic NSCLC tissue. Overexpression of SLC38A3 is responsible for EMT and cell motility in A459 cells and promotes metastasis formation in nude mice. The mechanism underlying this activity is a decrease in the level of glutamine (and histidine) with the simultaneous activation of the AKT pathway mediated by 3-phosphoinositide-dependent kinase 1 (3-PDK1). Researchers have suggested that SLC38A3 activity could be facilitated in an acidic environment when cells leave the primary tumor sites, thus, avoiding the unfavorable Na^+^ electrochemical gradient [180].

SLC38A3 ↑ ⇒ GLU (HIS) ↓ ⇒ 3-PDK1 ↑ ⇒ AKT↑ ⇒ EMT, cell motility, metastasis ↑

#### 6.2.3. ROS Metabolism

Aldehyde dehydrogenase 1 (ALDH1) is involved in the oxidation of toxic aldehydes that represent reactive carbonyl species (RCS) mainly derived from ROS-dependent lipid peroxidation and that are themselves capable of contributing to increased ROS.

High levels of ALDH1 lead to the acquisition of EMT/CSC properties and resistance to erlotinib in LC cells [181].

ALDH1 ↑ ⇒ EMT traits (VIM ↑, CDH1 ↓, migration ↑, stemness ↑, resist. to erlotinib ↑)

#### 6.2.4. Others

NAD levels are responsible for EMT in A549 cells. They are modulated by overexpression of CD38 (a NADase) and proteomic analyses have revealed significant metabolic rewiring in NAD-depleted cells. Many factors involved in EMT are upregulated by NAD depletion and, in particular, three pathways are involved, i.e., the integrin/AKT1/β-catenin, the IL6/JAK1/STAT3, and the TGFβ/p38 pathways. Interestingly, NAD depletion has led to the secretion of factors involved in EMT (such as TGFβ) that could have a paracrine effect within the TME. Importantly, supplementation with NAD precursors (i.e., nicotinamide and nicotinic acid) reverses the effect of CD38-dependent depletion of NAD [182].

NAD ↓ ⇒ integrin/AKT1/β-catenin ↑, IL6/JAK1/STAT3 ↑, TGFβ/p38 ↑ ⇒ EMT ↑

MTHFD2 (methylentetrahydrofolate dehydrogenase 2) is involved in one-carbon metabolism participating in several biosynthetic pathways related to the utilization of formate. Interference with MTHFD2 has been observed to have relevant effects in terms of cell proliferation, motility, invasiveness, and also towards the EMT process. However, the metabolism-related mechanism was not clearly demonstrated [183]. Indeed, proteins involved in metabolism often have “moonlighting” functions; in particular, MTHFD2 has been involved in RNA processing and translation by direct interaction with nuclear factors involved in RNA metabolism and translation [184], and has recently been involved (albeit in renal cell carcinoma) in the M6A methylation of HIF-2α mRNA, thus, contributing to its expression and to the promotion of aerobic glycolysis [185].

MTHFD2 ↑ (moonlighting function) ⇒ AKT/GSK3β/β-catenin ↑ ⇒ EMT

When the EMT process is induced in LC A549 cells, morphological, numerical, functional alterations of mitochondria (swelling, loss of cristae, reduction in the number and loss of respiratory properties), and a shift towards anaerobic glycolysis are clearly observed. TGFβ1 affects mitochondria by the downregulation of SIRT1 (silent information regulator 1), but the most interesting finding is that SIRT1 forced-expression prevents TGFβ1-induced mitochondrial misregulation and EMT itself, thus, highlighting a central role of mitochondria metabolism integrity in the regulation of EMT [186].

TGFβ1 ↑ ⇒ SIRT1 ↓ ⇒ mitochondrial disfunction ⇒ EMT

Intriguingly, a reduction in the activity of glycolysis, TCA cycle, lipid synthesis, and ROS signaling has been observed in LC cells in which EMT was induced. The researchers demonstrated that these alterations were linked to the expression levels of Nrf2, in particular, a decrease in Nrf2 is associated with both a metabolic slowdown and the mesenchymal phenotype. Researchers have raised an interesting consideration regarding the changes in metabolism from primary tumor, migrating cells, and cells that colonize the metastatic niche, in which a sort of EMT-MET occurs. EMT and MET in metastasis formation is a theme that has been deeply discussed [187] and a deeper comprehension of the associated metabolic alterations could hopefully provide important insight to counteract cancer cell dissemination [188].

### 6.3. Thyroid Cancer

**Figure 4:**
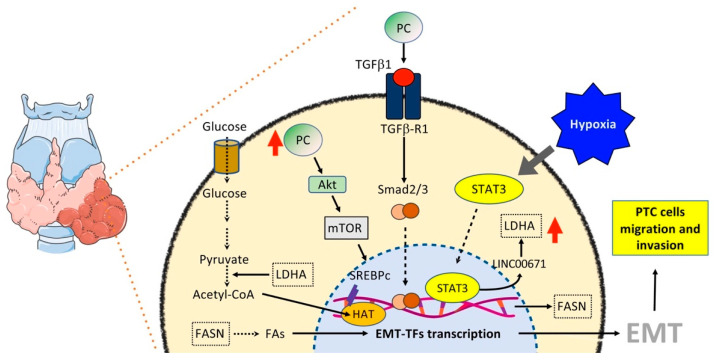
Overview of some of the main mechanisms connecting metabolism and EMT in TC.

Thyroid cancer (TC) represents the most common endocrine malignancy, with an increasing incidence all over the world. Papillary TC (PTC), a differentiated TC (DTC) subtype, is the most common and, even though it has an excellent prognosis following radioiodine (RAI) ablation, it shows an aggressive behavior in 20–30% of cases, displaying loss of differentiation features, including loss of the expression/function of the sodium iodine symporter, with consequent RAI treatment failure and high mortality. In addition, anaplastic thyroid carcinoma (ATC), the most undifferentiated TC, is a rare but devastating disease. EMT plays a pivotal role in TC progression, as a differentiated TC could progress to ATC as a result of either a dedifferentiation process or the development of CSCs, and both depend on EMT [189]. Metabolic changes are also critical in TC progression. Accordingly, overexpression of HIF-1α, as well as HK2, phosphoglycerate kinase (PGK), G6PDH, LDHA, GLUT1, and monocarboxylate transporter 4 (MCT4) has been observed in TC, associated with distant metastasis [189]. Overall, we can observe, so far, that there is increasing evidence that metabolic changes may be responsible for EMT in TC but not yet vice versa (Figure 4). Specific in vitro and in vivo experiments, as detailed below, have investigated the mechanism linking metabolic reprogramming to EMT in TC progression. 

#### 6.3.1. Glucose and OXPHOS Metabolism

The interplay between EMT and metabolism in PTC progression toward a metastatic stage has been showed by the observation that expression of pyruvate carboxylase (PC), the enzyme that catalyzes the carboxylation of pyruvate to form oxaloacetate, is higher in PTC tissues and thyroid FNA washout fluid samples from patients with central lymph nodes metastases than those without them, and that PC-induced PTC metastases may occur through TGFβ-mediated EMT (Figure 4). In particular, silencing of PC inhibits EMT by reducing the protein expression of P-Smad2/3 and TGFβR1 [190].

PC ↓ ⇒ TGFβ/Smad pathway ↓ ZEB1, SNAIL ↓ ⇒ EMT ↓

In another recent study, higher expression levels of LDHA, the enzyme that catalyzes the last step of the Warburg effect by converting the pyruvate and NADPH to lactate, were correlated to PTC progression. Briefly, LDHA is activated during hypoxia through activation of STAT3, which, in turn, inhibits the long non-coding RNA LINC00671, resulting in induction of LDHA. This axis regulates PTC glycolysis, growth, and metastasis, suggesting that LDHA could induce EMT (Figure 4) [191].

Hypoxia ↑ ⇒ STAT3 ⇒ LINC00671 ↓ ⇒ LDHA ↑ ⇒ EMT

The gap between LDHA and EMT was bridged by a study showing that high levels of LDHA lead to increased levels of acetyl-CoA, which increased the histone acetylation of EMT-related genes, including *CTNNB1*, *RHOB*, and *TGFβR1*, thus, promoting EMT (Figure 4) [192].

LDHA ↑ ⇒ acetyl-CoA ↑ ⇒ CTNNB1, RHOB, TGFβ R1 ↑ ⇒ EMT

However, a small study on nine patients, reported increased expression of the circular RNA circCCDC66 in TC samples as compared with normal tissue, which supported a role for enhanced levels of PDK1, the enzyme that acts to inactivate the pyruvate dehydrogenase complex, thus, inhibiting the conversion of pyruvate to acetyl-CoA and, instead, increasing its conversion to lactate in the cytosol, in TC progression. Briefly, circCCDC66 enhances migratory and invasive capacities of TC cell lines by sponging miR-211-5p, which, in turn, targets PDK1 [193].

circCCDC66 ↑ ⇒ miR-211-5p ↓ ⇒ PDK1 ↑ ⇒ EMT ⇒ cell migration and invasion ↑

A group of studies has shown that sirtuin 6 (SIRT6), which is upregulated in PTCs and associated with tumor progression, induces both the Warburg effect through activation of ROS, and EMT through upregulation of HIF-1α, which in turn upregulates the expression of SNAIL and TWIST [194,195,196].

SIRT6 ↑ ⇒ ROS ↑ ⇒ HIF-1α ↑ ⇒ SNAIL, TWIST ↑ ⇒ EMT

⇓

PKM, GLUT1, HK2, LDHA, Eno1, PGK1, GAPDH ↑ ⇒ Warburg effect

#### 6.3.2. Lipid Metabolism

Cholesterol molecules and their metabolites play important roles in normal cells, because they participate in the formation of membranes, and are particularly required by tumor cells, which increase their uptake from the bloodstream or activate de novo cholesterol biosynthesis to sustain their growth and development. In a cohort of patients with benign thyroid tumors, PTC (low/intermediate and high risk), poorly differentiated (PDTC), and ATC, the patients with more aggressive tumors (high-risk PTC, PDTC, and ATC) showed decreased levels of blood LDL cholesterol and apolipoprotein B associated with an increase in intra-tumor 27-hydroxycholesterol (27-HC) and a decrease in the mitochondrial enzyme 25-hydroxycholesterol 7-alpha-hydroxylase (CYP7B1), which is responsible for 27-HC catabolism. LDL cholesterol promotes proliferation and migration, while CYP7B1 overexpression arrests growth and decreases migration of ATC cell lines, suggesting that cholesterol and intra-tumor accumulation of 27-HC promote progression of PTC [197].

CYP7B1 ↓ ⇒ 27-HC ↑ ⇒ EMT

An increase in FA levels was observed as a further consequence of PC upregulation in PTC associated with tumor progression. In particular, PC induces the Akt/mTOR signaling pathway, thus, upregulating SREBP1c, which, in turn, upregulates FASN and, consequently, lipogenesis and EMT (Figure 4) [198].

PC ↑ ⇒ SREBPc ↑ ⇒ FASN ↑ ⇒ FAs↑ ⇒ ZEB1, SNAIL ↑ ⇒ EMT

Consistently, by crossing proteomic with the metabolomic data of ATC cells that have previously been shown to be induced to MET following overexpression of the transcriptional factor PATZ1 [199], drastic downregulations of PC, FASN, and FAs as compared with backbone vector-transfected cells have been observed [200]. This also suggests that PATZ1 could be upstream of the above pathway, and is likely involved in the downregulation of PC expression. 

## 7. Therapeutic Perspectives

Targeting cancer metabolism is an attractive anticancer therapeutic approach that has recently started to be developed. So far, there has been interest in glutamine, asparagine, adenosine and lipid synthesis, as well as glycolysis and the TCA cycle [201]. Three drugs, based on targeting asparagine metabolism in acute lymphoid leukemia and mutant IDH1/2 in acute myeloid leukemia, have received approval from the U.S. Food and Drug Administration (FDA), and many others are under development either in preclinical or clinical studies [201]. As we have shown in the previous sections, the interplay between EMT and metabolism is tightly associated with the metastatic process, envisaging the reversion of EMT, through the targeting of specific metabolic enzymes or metabolism-dependent epigenetic reprogramming, as a way to limit tumor progression. Inhibition of glutaminolysis by targeting GLS1 or GLS2, for example, reduces LC and hepatocellular carcinoma (HCC) metastases, respectively, through repression of SNAIL [202]. Among GLS inhibitors, CB-839 has been shown to be effective against TNBC and hematological tumors [203] and is currently undergoing clinical trials [72].

Other studies have shown that by targeting lipogenesis it is possible to inhibit EMT and, consequently, tumor progression. Indeed, the FASN inhibitor TVB-2640 is under clinical trials to test its efficacy, alone or in combination with paclitaxel, in inhibiting tumor growth and metastasis of different cancers [204], while the inhibition of peroxisome proliferator-activated receptor family members, regulators of FAs synthesis and oxidation and involved in both lipid metabolism and EMT inhibition, induces metastasis in melanoma and increases stemness in prostate cancer [205,206]. Recently, the metabolism of sphingolipids has aroused much interest in EMT and tumor progression in HCC. Its disruption, by inhibiting sphingosine kinase 1 (SPHK1), which induces EMT through autophagic degradation of E-cadherin [207], or its derivate sphingosine 1-phosphate (S1P), which induces EMT through the matrix metalloproteinase-7 (MMP-7)/TGFβ autocrine loop [208], could be considered to be promising therapeutic approaches against HCC metastasis [201]. Glycolytic metabolism in fast-growing solid tumors is enhanced by the hypoxia-induced HIF-1α, which, in turn, promotes EMT and metastasis [209,210]. Therefore, it may be targeted by HIF-1α inhibitors, such as the antisense oligonucleotide EZN-2968, which, in a pilot phase I clinical trial, has been shown to modulate HIF-1α and its target genes in biopsies of refractory solid tumors [211]. Still referring to glucose metabolism and its intersection with EMT, some preclinical studies are under way to test the efficacy of GLUT inhibitors, such as WZB117 and the natural flavonoid silibinin, as anticancer drugs [72]. Indeed, GLUT3 expression correlates with EMT-TFs in NSCLC [171]. Acidity, which is a consequence of the Warburg effect and lactate accumulation, is another crucial cancer feature that different studies are trying to exploit for the development of anticancer strategies, since it has been shown to induce EMT and metastasis [212]. However, cancer cells must neutralize excess intracellular acidity to survive. Several molecules are involved in the regulation of acidity, including the carbonic anhydrase IX (CAIX), a membrane bound isoform of CA transcriptionally regulated by HIF-1α. It helps the cancer cell to neutralize intracellular acidity by inducing extracellular acidification catalyzing CO_2_ hydration. An acidic environment can be tolerated by tumor cells, but it inhibits growth of normal cells, thus, facilitating invasion and metastasis [213]. Therefore, it allows cancer cells not only to survive in a condition of lack of oxygen, but also to become more aggressive and spread throughout the body. Indeed, CAIX overexpression is associated with poor survival in BC patients [214]. This upregulation appears to be a consequence of EMT, as a way to compensate for the downregulation of the other carbonic anhydrase member CAXII following PMA-induced PKC activation in BC cells. Interestingly, PMA treatment also induces upregulation of LDHA and HK2, supporting the functional interaction between metabolic enzymes and transporters in mediating EMT cellular effects [215]. In addition, CAIX has been shown to drive the stromal-induced EMT in prostate cancer and protect cells from ferroptosis [216,217]. Inhibition of CAIX counteracts EMT CAF-induced invasion and metastasis [216], and enhances ferroptotic cell death [217]. Recently, the CAIX inhibitor SLC-0111, has entered anticancer clinical trials [218]. Previously, it has been shown to efficaciously block CAIX and tumors in experimental animal models of melanoma, breast, brain, and pancreatic cancer [219,220,221]. 

## 8. Conclusions

EMT plays a key role in triggering both the process of cellular invasion and metastasis and the acquisition of chemoresistance. The capacity to adapt in different environmental conditions or in the presence of chemotherapeutics is the main characteristic of aggressive tumors and is tightly linked to EMT, stemness, and metabolism rewiring. Growing evidence demonstrates an interplay between EMT and metabolic changes. In particular, the fact that metabolism can positively influence the promotion of EMT has led to hypothesize possible therapeutic approaches based on the targeting of specific metabolic enzymes to counteract the induction and advancement of metastases. Some of them are either available or under investigation for therapeutic strategies in different tumors. The critical challenge is to identify the time window useful for counteracting EMT during tumor progression, to avoid inducing MET in cells that have already left the primary site, which would favor their colonization of a secondary (metastatic) site.

## Figures and Tables

**Table 1:** LncRNAs involved in EMT and Warburg effect.

LncRNA	EMT	Warburg Effect
**PROMOTERS**		
HOTAIR	Activates SNAIL by recruiting PRC2 on miR-34 promoter, which, in turn, targets SNAIL [101] Represses E-cadherin and other SNAIL targets by recruiting EZH2 on their promoters [102] Acts as ceRNA that positively regulates SNAIL2 by sponging miR-148a [103]	Acts as ceRNA that positively regulates HK2 by sponging miR-125 and miR-127 [104] Upregulates glucose transporter 1 (GLUT1) via mTOR [105]
MALAT1	Acts as ceRNA that positively regulates Slug by sponging miR-204 [106] and Capn4 by sponging miR-124 [107] Represses E-cadherin by recruiting EZH2 on its promoter [108]	Stabilizes HIF-1a by promoting its dissociation from VHL and thus preventing its ubiquitin-dependent degradation [109]
H19	Acts as ceRNA that positively regulates EMT genes, including vimentin, ZEB1/2, HMGA2, and others, by sponging miRNAs targeting them [110,111] Activates β-catenin/GSK3β signaling by recruiting EZH2 [112]	Activates tumor specific PKM2 [113] Acts as ceRNA that positively regulates PDK1 by sponging let-7, thus causing the release of HIF-1a [114]
UCA1	Acts as ceRNA that positively regulates ZEB1/2, SNAIL2 and HMGA2 by sponging miR-145, miR-203, and miR-485-5p, respectively [115,116,117] Enhances Wnt/β-catenin signaling [118]	Upregulates HK2 through the mTOR-STAT3/miR-143 pathway [119]
TUG1	Acts as ceRNA that positively regulates ZEB1/2, by sponging miR-145 [120,121], and EZH2, by sponging miR-382 and miR-144-3p [122,123]	Upregulates HK2 through the TUG1/miR-455-3p/AMPKβ2 axis [124]
PVT1	Acts as ceRNA that positively regulates PTCH1, thus activating hedgehog signaling, by sponging miR-152 [125]	Acts as ceRNA that upregulates HK2 by sponging miR-497 [126]
ANRIL	Activates ATM-E2F1 signaling [127]	Upregulates GLUT1 through the PI3K/AKT/mTOR pathway [128]
CRNDE	Enhances Notch signaling [129]	Upregulates GLUT4 [130]
ROR	Acts as ceRNA that upregulates EMT TFs including Nanog and ZEB2 by sponging miR-145 and miR-205 [131,132] Activates ZEB1 by inhibiting p53 [133]	Upregulates HIF-1α by sponging miR-145 [134]
CASC9	Acts as ceRNA that upregulates TGFβ2 and EGFR by sponging miR-758-3p and miR-370, respectively [135,136]	Interacts with HIF-1α and stabilizes its protein [137]
**INHIBITORS**		
LincRNA-p21	Inhibits Notch and Hippo signaling [138,139]	Competes with HIF-1α for the binding to VHL [140] Downregulates PKM2 [141]
GAS5	Acts as ceRNA that upregulates aplasia Ras homologue member I (ARHI) by sponging miR-221 [142]	Inhibits the expression of 6-phosphoglucanase (G6Pase) and phosphoenolpyruvate carboxykinase (PEPCK) [143]
